# A qualitative exploration of experiences of gender identity and gender questioning among adults with Klinefelter syndrome/XXY

**DOI:** 10.1002/jgc4.1952

**Published:** 2024-07-22

**Authors:** Claire Harkin, James Elander

**Affiliations:** ^1^ University of Derby Derby UK

**Keywords:** gender identity, gender questioning, Klinefelter syndrome, testosterone

## Abstract

People with Klinefelter syndrome (KS/XXY) may be at higher risk of gender dysphoria than the general population and gender diversity needs greater recognition and consideration in services for people affected. This study aimed to give systematic insights into experiences of gender diversity among people with KS/XXY, which could inform more person‐centered care for people with KS/XXY and contribute to practical guidance for healthcare professionals. We conducted individual, semi‐structured interviews with 11 adults with diagnosed KS/XXY. The verbatim interview transcripts were analyzed using experiential reflexive thematic analysis, which identified four themes: (1) *Experience of gender*, which described participants' experiences of exploring and negotiating their gender identity; (2) *Navigating expectations*, which described how participants' gender uncertainty was associated with confusion, isolation, and shame, and how fears about other people's reactions caused participants to keep their gender identity secret; (3) *Testosterone assumptions*, which described how participants needed more discussion and counseling before testosterone replacement therapy (TRT), and how some benefited from treatment with alternative hormones to testosterone; and (4) *A different approach*, which described participants' experiences of care at gender identity clinics. The findings give new insights into the gender identity journeys of people with KS/XXY, from early attempts to understand and make sense of gender, through dealing with social pressures, the development of gender identities more congruent with feelings, and experiences with hormone replacement therapy. The practice implications include that there should be improved consideration of gender identity in care for KS/XXY, better psychological support for those affected by gender diversity, and more consideration given to alternatives to testosterone‐based therapies. Future research could explore the experiences of gender identity among different groups of people with KS/XXY, the development of gender identity over time, the effects of TRT on gender identity, and healthcare providers' knowledge and attitudes about gender identity and KS/XXY.

**What is known about this topic**

Many people with Klinefelter syndrome (KS/XXY) question their gender identity and some may be affected by gender dysphoria.

**What this paper adds to the topic**

This paper provides insights to inform better consideration of gender identity and gender questioning by healthcare professionals providing care for people with KS/XXY.

## 1 INTRODUCTION

Klinefelter syndrome (KS/XXY) affects approximately one in 660 births assigned as male; prevalence estimates range from 0.02% to 0.22% (Bojesen et al., 2003; Herlihy, Halliday, et al., 2011). However, up to 75% of cases remain undetected because the visible characteristics often associated with XXY, including gynecomastia, tall stature, and sparse body and facial hair, are highly variable and many people with KS/XXY are asymptomatic (Gravholt et al., 2018; Herlihy, Halliday, et al., 2011). The term “Klinefelter syndrome” usually refers to people with an XXY “karyotype” (set of chromosomes) who develop characteristics typical of Klinefelter syndrome and identify as male, whereas the term “XXY” usually refers to individuals with that same karyotype who either do not have those characteristics or do not identify as male. Some people view the term Klinefelter syndrome to be pathologizing and some do not. In this paper, we use the term “people with KS/XXY” to refer to all those groups of people.

The XXY karyotype is detected most frequently between 30 and 40 years of age, often during investigations for infertility (Groth et al., 2013). The advent of cell‐free DNA (cfDNA) testing for sex chromosome aneuploidies has made prenatal detection possible (Samango‐Sprouse et al., 2017; Shear et al., 2023), although cfDNA testing is presently available only among the relatively privileged in certain countries and is not yet common practice in the United Kingdom. However, early diagnosis could become more common as access to cfDNA testing increases and if routine post‐natal screening for KS/XXY is introduced. The most common treatment for hypogonadism is testosterone replacement therapy (TRT; Groth et al., 2013). However, testosterone will only treat testosterone deficiency whereas Klinefelter syndrome is multimodal; some features reflect genetic changes or a combination of testosterone deficiency and genetics.

KS/XXY may present multiple challenges to psychological adjustment and quality of life. In one survey of people with KS/XXY, subjective well‐being, body image, self‐esteem, mental health, social support, and general health were all much poorer than for the general male population (Herlihy, McLachlan, et al., 2011). In another, people with KS/XXY had lower educational attainment, income, physical activity, and both mental and physical quality of life than matched controls (Skakkebæk et al., 2018). In another, 68.8% of people with KS/XXY reported clinically significant depressive symptoms (Turriff et al., 2011).

There is also evidence that people with KS/XXY may be more likely to be gender divergent. In one questionnaire survey of 87 people with KS/XXY, only 65% reported feeling masculine whereas 30% reported feeling neither masculine nor feminine and 5% reported feeling feminine (Herlihy, McLachlan, et al., 2011). In another survey of 81 people with KS/XXY, 92.5% were recorded as male on their birth certificate but only 65% identified as male and only 61.64% said they enjoyed living as the sex recorded on their birth certificate (Cai & Yap, 2022). In another survey, people with KS/XXY had more symptoms of gender dysphoria (GD) than a control group of healthy males, but they had fewer symptoms than a group of people with “MtF GD diagnosis” (p. 2415) and none of the KS/XXY group had symptom scores indicating diagnosable gender dysphoria (Fisher et al., 2015). The etiology of gender diversity is not yet understood but suggested potential contributory factors among people with KS/XXY include reduced exposure to androgens (Chang et al., 2015) and characteristics like gynecomastia and sparse body hair, which could potentially make those affected more susceptible to doubts about a masculine identity (Tunas et al., 2017; see also Liang et al., 2022).

Numerous case reports described individuals with KS/XXY who were assessed or treated for gender dysphoria, sex reassignment, or other aspects of gender diversity. Some of those illustrated how testosterone improved mental health and reduced gender dysphoria (Korchia et al., 2018; Nishikawa et al., 2019; Tunas et al., 2017). Other case reports described people with KS/XXY who disliked the masculinizing effects of testosterone because they identified as female or were prescribed estrogen to promote the development of female sex characteristics, and those reports also illustrate the stigmatization, marginalization, and discrimination experienced by transgender and gender diverse people (Davies & Parkinson, 2018; Moustafa, 2017; Seifert & Windgassen, 1995).

However, quantitative surveys do not capture people's lived, phenomenological experiences of gender identity and gender questioning, and case reports present specific individuals who were selected and described in different ways, usually by their doctors. To better understand experiences of gender identity and gender questioning among people with KS/XXY, therefore, qualitative research is needed with groups of people who meet the same inclusion criteria, are investigated in the same way, and describe their experiences in their own words. The results could potentially inform the development of person‐centered care, advice, information, and support for people with KS/XXY. The aims of the present study were therefore to explore how gender identity and gender questioning are experienced by people with KS/XXY and to provide insights into those experiences that could inform the development of care for people with KS/XXY.

## 2 MATERIALS AND METHODS

### 2.1 Design

The study was conducted from a phenomenological perspective, with data generated by individual semi‐structured interviews and analyzed using experiential reflexive thematic analysis, which is “concerned with exploring the truth or truths of participants' contextually situated experiences, perspectives and behaviors” (Braun & Clarke, 2022a, p. 8). The Singular Framework for Methodological Integrity (Levitt et al., 2017) and the Consolidated Criteria for Reporting Qualitative Research (Tong et al., 2007) were followed to ensure fidelity and utility. The study protocol was approved by the University of Derby Research Ethics Committee (Reference ETH2122‐1547).

### 2.2 Participants

The participants were adults with KS/XXY. The inclusion criteria were having a confirmed diagnosis of KS/XXY, being 18 years or older, and being able to give informed consent. The exclusion criterion was having a known psychological condition that could put participants at risk of psychological distress. Participants were recruited via the Klinefelter's Syndrome Association, the XXY Project, and various KS/XXY Facebook support groups.

### 2.3 Procedure

Trustees and administrators of the Klinefelter's Syndrome Association, XXY Project, and KS/XXY Facebook support groups gave permission to post invitations to participate on websites and social media platforms. The invitations explained that the study was to explore the experience of gender questioning among individuals with Klinefelter syndrome (KS) and the impact this may have had on their well‐being, and they included a link to the participant information sheet and consent form, hosted by Qualtrics, a secure online survey platform. Participants who consented were contacted by email to schedule interviews. No incentives were offered.

Interviews were conducted via video call and were audio recorded and transcribed verbatim. Participants were asked to ensure privacy so they could talk openly. Each participant was interviewed only once. The interview schedule is given in Appendix S1 and was developed, reviewed, and pilot‐tested with a trustee of the Klinefelter's Syndrome Association (who was not a participant in the study) before the study began. The pilot test resulted in a few small changes in wording to improve clarity. In the interviews, participants were asked about the circumstances of their diagnosis with KS/XXY; their gender identification, and any confusion or dissatisfaction they felt about that; about any support they had received; about any hormone replacement therapy they had received and their experience of that; and about any services or support they would like to be available. The interviews lasted between 30 min and 2 h and were all conducted by the first author, who also undertook the transcription. Field notes of key contextual information about participants were made immediately after the interviews and these were used to put interview data in context.

### 2.4 Analytic strategy

While recognizing the socially constructed nature of gender identity (Bussey, 2011), the aim was not to explore the construction of gender identity but to explore participants' experiences of gender identity and gender questioning and inform the development of person‐centered care. Our ontological approach to participants' experiences was a critical realist (Lincoln et al., 2017; Maxwell, 2012) and our epistemological approach was phenomenological (Sundler et al., 2019).

Before the data analysis began, all the transcripts were checked and verified and in two cases, participants were recontacted to clarify points of detail. The themes were derived entirely from the data and were not identified in advance. The analysis interpreted participants' experiences using their own words as far as possible. Codes were initially generated at a semantic level and then themes were derived from the shared meanings to tell participant stories. The data analysis was conducted manually using a system of notes and comments and followed recommended good practice for reflexive thematic analysis (Braun & Clarke, 2019, 2021, 2022b).

The first author led the data analysis and the authors worked independently and together to review and confirm the themes that were generated at each stage of the analysis. Demographic information was recorded individually for each participant and was used and presented in a purely descriptive way with no statistical analysis.

### 2.5 Reflexivity

The first author is a woman and the second author is a man, and both identify with genders congruent with their assigned sex at birth. At the time of the study, the first author was a postgraduate psychology student with Masters level training in qualitative research methods and the second author was a Professor of Health Psychology with extensive experience in qualitative health psychology research.

The first author has a family member with a KS/XXY diagnosis and is a trustee and volunteer for charities that support individuals with KS/XXY and their families. One of the participants was also a trustee of the same charity. Neither author had any prior relationship with any of the other participants. The second author had no personal or professional involvement with KS/XXY.

To manage the researchers' perspectives in data collection, reflexive strategies were used, including open, non‐leading questions, and allowing participants to ask questions and provide information they felt was relevant or important. Throughout the data analysis, the authors were conscious of their own role and involvement in meaning making, and consciously privileged the participants' perspectives while acknowledging and considering the influence of their own perspectives (Braun & Clarke, 2019). Preliminary findings were reviewed by a senior clinician at a multidisciplinary KS/XXY clinic.

## 3 DEMOGRAPHIC RESULTS

There were 20 people who responded to the invitation to participate, of whom nine did not confirm consent, one did not respond to emails about arranging interviews, and 11 were interviewed. Participant information is given in Table 1. Pseudonyms were used and some personal details have been changed to protect participants' anonymity. There were four participants who self‐identified as male, three who self‐identified as female, and four who self‐identified as non‐binary or gender fluid. In terms of ethnicity and socioeconomic status, the participants were predominantly White and middle class.

**TABLE 1:** Participant information.

Pseudonym	Gender identity	Preferred pronouns	Age band	Country of residence	Most recent care received
Alex	Non‐binary	They/them	70s	USA	None
Charlie	Female	She/her	70s	Canada	Gender identity clinic
David	Male	He/him	50s	UK	Endocrinologist
Joe	Gender fluid	They/them	60s	Canada	Gender identity clinic
Kevin	Male	He/him	40s	UK	Endocrinologist
Louise	Female	She/her	30s	UK	Gender identity clinic
Maria	Female	She/her	50s	Australia	Gender identity clinic
Max	Non‐binary	They/them	80s	USA	None
Ronald	Male	He/him	50s	UK	Endocrinologist
Stan	Male	He/him	50s	UK	Multidisciplinary KS/XXY clinic
Tom	Gender fluid	He/they	30s	UK	Endocrinologist

*Note*: Certain details have been changed to protect participants' anonymity.

## 4 THEMATIC RESULTS

There were four themes: (1) *Experience of gender*; (2) *Navigating expectations*; (3) *Testosterone assumptions*; and (4) *A different approach*. These are described below, together with representative quotations.

### 4.1 Theme 1: Experience of gender

The essence of this theme was the experience of gender, including the recognition and acceptance of stereotypically feminine traits, the external expression of those traits, and experiences of gender diversity. Looking back and reflecting on past experiences and feelings informed current gender identities, which varied considerably. Stan and Louise, for example, described strong and stable but very different gender identities:

I'm male, I've always regarded myself as male. (Stan)

I know I felt like a girl since I was about 4 years old. (Louise)

Having a stable male gender identity did not necessarily mean seeing oneself as a stereotypical male, however; participants who identified as male also described themselves as distinctive or different because they were XXY:

I've always grown up knowing that I was a boy and being a boy … I'm definitely male, I'm just an XXY male. (Kevin)

The difference associated with being XXY was viewed as an additional component of gender identity, as illustrated below:

As a child I always had a female component when I was growing up. I had an interior female friend. I didn't give her a distinct identity, but I always felt that she was there. (Max)

Max talked fondly of having an internal female companion who had always been present and who they felt comforted by. Tom, who was gender fluid, reflected on their childhood and realized that from an early age some of their interests were stereotypically feminine:

… there definitely would have been times when I was younger … young enough to be doing things that would be considered a girl's thing. (Tom)

Tom also implied that their gender expression was changeable over time (“… *would have been times* …”) and their retrospective gendering of childhood behaviors reflected others' views (“… *would have been considered* …”). Those stereotypically feminine behaviors were not obvious to them at the time but would have been to those around them, especially parents or caregivers.

Experience of gender also included current awareness of stereotypically female traits in the present, including among participants who identified as male:

… it [KS/XXY] makes me feel emotional, it makes me want to cry and I think that is more of a female trait … (Stan)

I would say on an emotional level … that I've got a very special way of communicating with females. So, I prefer to talk to females more than males, customers or people generally. (Kevin)

Stan described feeling emotional as a KS/XXY individual, and Kevin described feeling he had a “*special*” way of communicating with females that suggested an affinity or empathy with female emotions and communication. This may have been related to the earlier statement that “*I'm definitely male, I'm just an XXY male*.” Kevin also described feeling he had strong emotional intelligence, which he also attributed to being XXY. Recognition and acceptance of stereotypically female traits included feelings about clothes:

… like me and my partner go shopping and she's getting clothes, I'm more interested in her clothes than my clothes because I think men's clothes are totally boring. (David)

Gender acceptance took place over time, often following many years of feeling “wrong,” which may well reflect hostile or discriminatory social attitudes:

… because I am neither male nor female and have had these experiences of a long, long life trying to figure out what is wrong with me, to discover that there's nothing wrong with me, I just fit into a different sort of corner. (Alex)

Alex described having felt in the past there was something “wrong” with them but how, having found a personal identity they felt comfortable with and aligned with who they were as an individual, they accepted their gender identity as a variation with its place on the gender spectrum.

Experiences that were described as “gender dysphoria” or feelings of being in the “wrong body” were also part of participants' experiences of gender:

… I'm in my late 40s and 50s and I'm wandering around the neighbourhood, wondering ‘Gee, I wonder what it would be like to be a woman,’ that I was having gender dysphoria at that point in time. (Alex)

… I felt, actually am I in the wrong body? (Joe)

Alex wondered “*what it would be like to be a woman*” and Joe described feeling they were born into the “*wrong body*.” Charlie referred to the self‐loathing she felt toward herself, specifically her penis:

… that's how much I hated myself and the junk [penis] God gave me. (Charlie)

This theme gave insights into how participants with different gender identities, including stable male identities, recognized and accepted traits and behaviors they associated with females. It also captured ways in which gender identity was viewed over time, as participants looked back at their earlier selves and assimilated earlier experiences into adult gender identities, including feelings of gender dysphoria later in life.

### 4.2 Theme 2: Navigating expectations

A central concept of this theme was uncertainty about the fit between individuals and the world, and the difficulty of navigating external expectations about gender, including external disapproval and feelings of secrecy and shame. Joe described experiencing uncertainty about their gender identity but also about how they “*fitted in*” to the external world:

I wasn't sure where I fitted in. […] I get along more with females than I do males … (Joe)

Joe had struggled to understand where they “fit” with their gender at a time when there would have been little information and support for any variations other than male or female. Tom and David shared recent experiences of fluctuating gender identity:

I don't feel happy in my gender balance as a man, but it's not necessarily a full switch that I absolutely want to be a female either, you know? There's been a lot of figuring out the blending, you're not 100% sure. (Tom)

It's like a vector twist basically, with digital people living in a binary world because we're always changing. (David)

A key aspect of participants' gender uncertainty was that it was often associated with experiences of external disapproval:

Father [told me to] ‘stop doing that because it's a girl's thing, you must do X, Y and Z’, you know? (Tom)

… the trouble is, you knew it was wrong … it's like we are all stereotyped and as a bloke you're not meant to do it … it's sort of like doing it under the covers sort of thing … (David)

Through external influences and engagement with close family and society, participants internalized that how they felt about and expressed their gender was “wrong.” Tom's and David's more “feminine” interests were not accepted by their families, which led to them keeping their true feelings about their gender identity secret. Looking back, Max recognized the toxic influence of society (“the poison from outside”) and how that impacted on how they felt about their emerging non‐binary gender identity:

Inciting us to feel thoughts indeed not mine, not theirs, not his and not hers. In my 20s I sensed a ‘we’ in me and failed to understand the poison from outside … (Max)

External influences can have a significant impact on how an individual both internalizes and expresses their gender identity. Experiences of external disapproval were linked with fear about how other people and society would respond to diverse gender identities:

[My partner] said I lied to her all these years. So, I didn't lie to you, I was afraid. I experienced a whole lot of fear because this wasn't right. (Charlie)

Charlie was fearful of how her partner would react, and fearful that her partner would reject her because she was made to think by society that her gender identity was “wrong,” illustrating how expectations related to social attitudes can undermine close relationships. Maria also illustrated the impact of fear of social disapproval:

I mean, I did want to live my life as a woman at that time, but I was afraid of what other people or what society would think of me. (Maria)

Secrecy about gender identity and shame about not sharing questioning and concerns with others made navigating social expectations about gender identity much more difficult:

And there's an element of like … internalised shame going on … throughout my life I would be very open to anyone that was LGBT, but in myself having that feeling internally and not being able to speak to anyone about it, you're sort of like, almost being derogatory towards yourself … (Tom)

Tom felt they had been unable to talk openly about how they were feeling and, while being very accepting of others, found it difficult to be accepting of themselves, punishing themselves internally as they expected society to punish them. Those experiences illustrate how feelings of fear and isolation can result from not being able to talk about or receive support when experiencing gender questioning.

This theme gave insights into how uncertainty and questioning about gender was associated with confusion, isolation, and shame, with fear about external responses causing participants to keep their gender identity secret. Societal norms made it difficult for participants to discuss and explore gender identity questions, and the data showed how sensitive they were to ways they felt their families and society might respond if they were open about their gender identity.

### 4.3 Theme 3: Testosterone assumptions

The central meaning of this theme was that hormone replacement therapy had implications that participants were not fully aware of beforehand. This theme included some positive experiences of testosterone when the dose was right, but the masculinizing effects of testosterone had negative impacts on some participants' gender identity and well‐being. Participants wished for more opportunities for discussion and counseling before starting hormone replacement therapy, and for opportunities to consider alternatives to testosterone, such as estrogen. Some participants were prescribed testosterone based on a doctor's apparent perception that they were not masculine enough in appearance, and the doctor's assumption that they wanted to look and feel more masculine. In some cases, participants were not asked whether they were happy or not with how their bodies looked, and were prescribed testosterone without discussion about gender identity or how testosterone would affect that:

… the doctor just immediately proceeded to put me on testosterone and there was no discussion about what the testosterone would do, other than it would make a man out of me. (Alex)

Alex described how a doctor implied that testosterone would help to strengthen a male gender identity, without confirming that Alex identified as male or wanted treatment to “*make a man*” out of them. For people with KS/XXY who do not identify as male, increasing male secondary sex characteristics could impair well‐being and intensify gender dysphoria. Charlie also described how doctors appeared to make assumptions about her inner identity, not just her appearance:

… the doctor just assumed that I was male, that I wanted to do something to make a man out of me. And I did not understand. So I just said, okay, fine. You know, he suggested it, I agreed with it. Because I was willing to do anything. I knew for years that there was something wrong. (Charlie)

For Charlie as well as Alex, doctors apparently assumed they identified as male and that the treatment aim was to “*make a man*” of them, without knowing them, their gender identity, or their life experiences. Charlie's description also illustrated how vulnerable people with KS/XXY can be to suggestion and how it was only by looking back from a later perspective that she was able to see that this treatment choice was wrong. Participants also described not being informed about potential side effects before starting testosterone replacement therapy (TRT):

… the endocrinologist told me that the only solution was to go on Delatestryl [testosterone enanthate injection] … I wish I had more information at that point. (Joe)

Joe wished they had been given more information about Delatestryl, a testosterone product, before taking it. Some participants described positive benefits of testosterone therapy, but for Charlie, the testosterone also intensified feelings about being female when she still presented as male:

… it built up my self‐esteem and it built up my confidence […] I always felt female, but the testosterone intensified that, and I really couldn't get away from it. (Charlie)

Although testosterone had some positive effects on Charlie, it also strongly affected how she felt about her gender identity. Charlie's description above, and Alex's below, illustrated how testosterone had unexpected effects on how participants felt about their gender identity, and how they experienced social and cognitive changes resulting from testosterone therapy:

There was no discussion about how it would change my mind, how it changed my thinking, which it did … how it would change my, the way I perceived the world and interaction with the world […] So, educational wise, from an XXY perspective, once I started getting the testosterone my left brain kicked in. (Alex)

Alex highlighted that no one explained the cognitive effects that could result from taking testosterone. The hormonal changes altered their perception, which had negative implications for their close relationships and self‐perception. However, Alex also described how testosterone enabled them to access the analytical part of their brain, which they had never been able to do before. Kevin welcomed the increase in energy that testosterone brought, but struggled with the increase in libido:

… I felt a lot more energy […] during the day it makes me feel really lustful … but artificially so … It's … a bigger thing for me because I've got a [religious] conscience and you're not supposed to lust after or look at women. (Kevin)

Kevin, who was religious and married, welcomed having more energy on testosterone but found the increased “lustful” feelings uncomfortable. He believed testosterone increased his libido but also his desire to watch pornography and felt shame and guilt because he believed that watching pornography and how it made him feel was sinful.

Some participants described how testosterone had negative and irreversible effects on their gender identity, or how, after experiencing side effects, they would have preferred not to have had TRT:

… when you're growing up, testosterone has quite a lot of irreversible side‐effects, especially like with the voice or with like, body hair and stuff like that. (Louise)

At the time Louise was having TRT, she was also secretly experiencing gender dysphoria, and some of the masculinizing effects of testosterone had remained physically since she transitioned.

What I was experiencing was simply that the testosterone was not working out for me and then I rejected a male identity […] I would have loved to have had some counselling beforehand … (Maria)

Maria wished she had received counseling beforehand because her experience of TRT led to her rejecting a male identity. It is possible that Maria had not accepted her female gender identity prior to the TRT, which gave her the push needed to accept it. Reflecting on their experience of TRT, Alex concluded they would have preferred not to have it:

Nothing is what would have been best. (Alex)

This theme therefore included experiences of positive effects of treatment with testosterone (when the dose was right), but the masculinizing effects of testosterone had negative impacts on the gender identity and well‐being of some participants who would have liked more opportunity for discussion and counseling about the implications of treatment with testosterone. Some participants had experienced treatment with alternative hormones to testosterone and found that these aligned better with their gender identity:

I said, you know, I take DHEA [Dehydroepiandrosterone; a hormone that helps produce other hormones including testosterone and estrogen]; I don't use testosterone […] I did try testosterone and I didn't find it was any better, it was costing me 50 bucks a week and I said no, this is stupid. (Max)

Max found their own cost‐effective way of producing DHEA by buying DHEA capsules and mixing it with coconut oil so it could be applied topically. Other participants had positive experiences with estrogen:

I feel great on estrogen. (Charlie)

Estrogen helped Charlie to develop consistent upper body muscle, unlike when she was taking testosterone and muscle development would drop off a few days after she stopped working out.

If I was to redo, I would take estrogen instead … (Louise)

Louise had a difficult experience regarding gender dysphoria when she was taking testosterone (see earlier), which could have been avoided if she had been given the option to start with estrogen. Some participants experienced benefits from treatment with hormones more commonly used by people who identify as female. Louise would also have preferred to have taken estrogen rather than testosterone. Having been given testosterone while secretly experiencing gender dysphoria, the masculinizing effects were distressing.

A key aspect of this theme was therefore that participants experienced medical professionals as assuming they wanted to become more masculine and prescribing testosterone without a prior discussion about the person's gender identity.

### 4.4 Theme 4: A different approach

There were four participants who were seen at gender identity clinics (Charlie, Joe, Louise, and Maria) and one who was on a waiting list to be seen (Tom). These participants experienced gender identity clinics as offering a different approach from the care they had received previously, but a key aspect of this theme was that this was another stage in their journeys, not necessarily an endpoint that resolved all previous difficulties. For some participants, their first experience of gender identity clinics was the long waiting time, which led Louise to seek private treatment:

… if you have problems, you can't call them, if they're too busy, it's just overwhelming. So, in the end I went private. (Louise)

However, Tom used the waiting time to continue exploring their gender identity with the security of knowing that they were on a waiting list, in the hope that by the time they were seen, they would have a better idea of what they wanted:

There's an element of knowing that it's there and you can kind of figure yourself out, because I don't think I'm even necessarily ready for having that meeting right now. You know, I'm kind of in the figuring out period … (Tom)

A key element of participants' experiences at gender identity clinics was that they experienced the specialist professionals differently from those they had seen previously, and were very aware they were dealing with a different type of professional:

… this psychiatrist, he was basically the gatekeeper. He was the person that you had to see, there was only one person in the province. (Charlie)

Charlie had to convince the psychiatrist that she should be offered surgery, and she felt considerable pressure as she knew that the psychiatrist could grant or deny this request. For some participants, it was a relief to deal with specialist professionals:

At that time, I was referred to a different team of medical doctors; psychiatrists, endocrinologists and, you know, people who understood gender differences. (Maria)

Prior to attending the gender identity clinic Maria had met with other medical professionals who had not been able to give her the support or treatment she needed. It was reassuring for Maria and important to her that she was supported by gender specialists. However, the experience of meeting clinical specialists who did not make automatic assumptions about their gender could be dramatic and frightening in its own way:

… they sent me up to this clinic. It had good and bad, the, all the research people that are associated with the, the fellow who was running it, the PhD guy. I can't remember his name, but he scared the living shit out of me, and I think he may have hit the nail on the head, but, with his diagnosis […] And he just said, ‘You're this,’ he just went through it and sent me on my way. And I was blown up, I couldn't fathom, I couldn't get my head around it I, I was in quite a mess. (Joe)

Joe found that being treated by someone who did not automatically assume that Joe had a male identity was very different from what they had experienced previously but was also unnerving in its own way. Joe went on to describe how they reached the conclusion they were gender fluid:

Anyway, there's a lady there and she had, she was Frank was his, was his name. He was, had the gender, what do you call it, re … Did the operations. So he [be]came Frances [not their real names]. So anyway, but he was very helpful. […] And a couple of the PhD candidates. Actually, the two guys were very, very helpful. Also, they, they got me on to a bookstore in [redacted], which I bought a number of books on. It took me many years to, to realize that I probably should have gone the other way […] I was reading on the KSA site back when I was, I was having all these problems. I didn't, there was no definition around it. But I think I came across the word gender fluid. That's probably more where I am. (Joe)

Joe's experience makes the point again that it can take time to feel comfortable with a gender identity. One insight this provides is the importance of other people helping to get the information needed (“*… they got me to a bookstore* …”). Another is that the process took time and often made sense only in retrospect (“… *it took me many years to, to realize …*”).

A noteworthy contrast between Maria's and Joe's accounts is that whereas Maria felt relieved and reassured to receive care from “*people who understood gender differences*”, Joe had a “*good and bad*” experience; bad with the doctor, “*the PhD guy*,” but then a better experience with more junior staff or students at the clinic, who took the time and effort to explain, inform, and enable Joe to make up their own mind.

Some participants described how surgery did not resolve all their gender identity issues:

[After surgery] I didn't know how to express myself as a female. That was the person I wanted to be, but it's still, I still have feelings about being male. (Charlie)

Charlie described how she still struggled with identifying as a woman even after surgery, as her psychological transition required more time and specialist support. Despite her feelings of relief and reassurance at being treated by gender identity specialists, Maria felt that gender dysphoria may be experienced or perceived differently by people with KS/XXY compared with how it is experienced by non‐KS/XXY people considering transitioning or contacting gender identity clinics:

I don't think an XXY person's experience with gender dysphoria is the same as an XY person's experience with gender dysphoria, someone transitioning from male to female. (Maria)

Maria's and Charlie's experiences give insights into ways that people with KS/XXY may have subtly different care needs from other groups of gender diverse people. This theme gave insights into the challenging experience of being seen by health professionals with different approaches, and how new types of care raised new issues for participants. It also highlighted how participants believed their experiences of gender dysphoria were different from those of other non‐KS/XXY people, and how surgery did not necessarily resolve all their gender‐related concerns.

## 5 DISCUSSION

The findings gave insights into participants' gender identity journeys and showed how gender identity development took time, during which participants were vulnerable to external pressure from family and society and kept many feelings secret from partners and others. They also gave insights into ways that some aspects of treatment for KS/XXY, especially testosterone replacement therapy (TRT) complicated those journeys for some.

Many aspects of participants' gender identity journeys resembled those of people more generally who question their gender or transition from one gender to another, including the recognition and acceptance of preferences and traits perceived as feminine. The social difficulties experienced with family and social expectations, and the perceived need to keep true feelings secret, which can affect mental health (Bedrov & Leary, 2021), resembled to some extent the stigma and discrimination experienced by transgender people (Drabish & Theeke, 2022) and the “negative social consequences of gender identity,” which was an overarching theme in a meta‐synthesis of experiences of gender dysphoria among transgender people (Cooper et al., 2020). They are also consistent with evidence about how diverse gender identity development can affect well‐being and mental health (García Vega et al., 2018; Wilson, 2018), and about the need for transitioning counseling and support (Harrison et al., 2020).

A key aspect of the findings that was different from what is known about diverse gender identity development more generally was participants' experiences of hormone replacement therapy. The analysis captured experiences of being prescribed testosterone on the assumption that participants wished to be more masculine, which was often not the case, without discussion or exploration of the alternatives, and how this sometimes affected their gender identity development.

The analysis also captured both positive and negative experiences at gender identity clinics, where participants appreciated the different approach from the care they were used to but found it did not necessarily resolve all their issues. However, participants' experiences of clinics with limited resources and long waiting lists are consistent with evidence about experiences of gender identity clinics more generally; in one study the average waiting time for a first appointment with a gender identity clinic was 18 months and waiting times contributed to low mood, suicidal ideation and reduced quality of life (Henderson et al., 2022).

### 5.1 Practice implications

The study findings have potential implications for several aspects of care for people with KS/XXY, which is currently provided through different services. Multidisciplinary clinics aim to provide comprehensive, integrated care, and there are presently 13 such KS/XXY clinics in the United States, two in the United Kingdom, and one in Denmark (https://genetic.org/). However, a common care pathway is for a general or family practitioner to arrange genetic testing and then refer the person to a general endocrinologist if KS/XXY is confirmed, so standards of care may vary and there is no detailed practical guidance for GPs, endocrinologists and other healthcare providers about how to approach and handle issues related to gender identity among people with KS/XXY.

#### 5.1.1 Considering gender identity issues more routinely

More research is needed, but the present findings suggest that people with KS/XXY would benefit from opportunities to explore potential gender identity issues in a more routine way, without needing to raise the issue or express a wish to begin transitioning. This would help with the implementation of existing recommendations for the care for people with KS/XXY, which include “considering psychosexual and psychiatric issues in all adult patients with KS” and giving “attention to the putative existence of gender incongruence in patients with KS” (Zitzmann et al., 2021, p. 157). An obvious opportunity for this might be the transition from pediatric to adult care, or the first clinical appointment in adult care, which would also address the recommendation about “improving the transitional care for patients with KS from paediatric to adult endocrinologists/andrologists” (Zitzmann et al., 2021, p. 158). Including gender identity issues in patient information and education materials could also help, possibly with case studies to illustrate the range of possible experiences, or materials modeled on the “What we wish” and “Know your rights” resources produced by InterACT (https://interactadvocates.org/resources/intersex‐brochures/).

#### 5.1.2 Improving knowledge and awareness of KS/XXY

Many of the present participants were not diagnosed until relatively late in life, but there is now greater recognition of the value of early testing and population screening for KS/XXY (Chromodiversity Foundation, 2024; Herlihy & McLachlan, 2015). The present findings also suggest that healthcare professionals should consider the social pressures and vulnerabilities that people with KS/XXY may experience, to ensure their care is not based on mistaken assumptions, and that care for KS/XXY acknowledges social discrimination against gender diverse individuals. This could help to implement the recommendation about “improving knowledge about KS among doctors and society” (Zitzmann et al., 2021, p. 158). Our participants' descriptions of medical professionals' assumptions might have reflected wider societal norms or a medicalized approach in which reduced testosterone was perceived as a symptom for which the obvious treatment was testosterone replacement. In either case, better training for health professionals could help improve this aspect of care for KS/XXY.

#### 5.1.3 Hormone replacement therapy

Many people with KS/XXY benefit from TRT and welcome the masculinizing effects (Chang et al., 2020). The benefits of TRT for people with hypogonadism are improved bone density, body composition, mood, erythropoiesis, cognition, quality of life, and cardiovascular health (Bassil et al., 2009). It is important therefore not to reduce access to TRT when it is appropriate, but our participants' experiences suggested that testosterone affected them in different and unexpected ways, especially those experiencing gender questioning or gender dysphoria prior to starting TRT. Their experiences illustrated the complexities of human responses to hormone treatments and the potential harms of automatically assuming TRT is the right therapy for KS/XXY, and they support recommendations for more open discussion about TRT and more careful monitoring of people's responses to it, consistent with other emerging evidence about experiences of testosterone therapy among people with KS/XXY. In one survey of 114 people with KS/XXY, 70% were receiving TRT and 12% were receiving estrogen, but only 55% identified as male and “only a third of individuals receiving HRT seemed satisfied with the physical changes they saw” (Kago et al., 2023, p. e1032). Those and the present findings suggest that endocrinologists and other practitioners should explore potential gender identity issues before prescribing testosterone or other hormone therapies for people with KS/XXY, and that alternative hormone therapies to testosterone should be considered. It is also worth considering the potential issues with alternative hormones, however, because the risks of venous thrombotic events, osteoporosis, and breast cancer are all increased by estradiol, a feminizing hormone often prescribed for trans people seeking hormonal treatment. One review concluded that “overall, it is important that gender‐affirming treatment is appropriately modified according to the risks and medical implications of Klinefelter syndrome. Furthermore, trans individuals should be informed and counselled about the unique risks and different options for feminizing gender‐affirming hormone therapy before initiation of treatment.” (Liang et al., 2022, p. 9).

Just as people with KS/XXY should not be pressured into accepting TRT, it is also important that people with KS/XXY should not be actively encouraged or pressured to make other decisions about gender identity that could have long‐lasting consequences. Some people with Klinefelter syndrome may be vulnerable to misconceptions around sex chromosome aneuploidies and the stigmatization of intersex conditions and gender diversity, and surveys suggest that self‐esteem, mental health, social support, and educational achievement are all lower on average among people with KS/XXY (Herlihy, McLachlan, et al., 2011; Skakkebæk et al., 2018; Turriff et al., 2011). Rates of language and communication difficulties were higher among children with Klinefelter syndrome and other sex chromosome trisomies (Bishop et al., 2011), and young people with KS/XXY scored lower on tests of executive function including verbal fluency, working memory and spatial planning, compared with control groups (Lee et al., 2011). Adults with KS/XXY had poorer social cognitive processing and emotion regulation than control groups and general population norms (Van Rijn et al., 2006; van Rijn & Swaab, 2020). In a large national study, people with Klinefelter syndrome were more likely than general population controls to be on the autistic spectrum and to have attention deficit hyperactivity disorder as well as being at increased risk of schizophrenia and bipolar disorder (Cederlöf et al., 2014).

As noted in the introduction, people with KS/XXY vary considerably in the ways and extent to which they are affected, but the increased likelihood of neurodiversity and the increased risk of difficulties with executive function, social cognition, emotion regulation, and other adversities could make it more difficult than otherwise for people with KS/XXY to articulate their feelings and wishes and negotiate treatment and care plans. Those factors mean that health professionals discussing gender identity issues with people with KS/XXY should be mindful of other potential factors that could influence their mental health and well‐being.

Hormone replacement therapy may be an aspect of KS/XXY care that could be improved by following good practice guidelines for the assessment and treatment of gender dysphoria among adults more generally. These recommended that: “Treatment must be patient‐centred and should recognise the individual's preferences, needs and circumstances. Treatment must not be prescriptive and should allow clinically safe choices for individuals. Patients should be accorded a substantial role in determining the kinds of treatments that are appropriate for them.” (Wylie et al., 2013, p. 19). They also recommended that: “Persons with gender dysphoria have a right to counselling and psychotherapeutic practice as part of the overall package of care,” (Wylie et al., 2013, p. 10) and that, “Psychological therapies should be available to be used as part of the patient's treatment programme. It should enable people, through a variety of approaches, to be clearer about their gender identity including whether they want to commence, continue, or reverse treatment.” (Wylie et al., 2013, p. 23). Those principles could usefully be incorporated in care for KS/XXY, so that people seen in endocrinology departments and KS/XXY clinics have the same opportunities for discussion and counseling before decisions are made about treatment, especially hormone treatments.

#### 5.1.4 Specialist gender identity services for people with KS/XXY

Maria's view that an XXY person's experience of gender dysphoria may be different from that of other people transitioning to become trans women might suggest that gender identity assessment and treatment approaches should be tailored for people with KS/XXY, and that practitioners in gender identity clinics should have a greater appreciation of how a KS/XXY person's experience of gender diversity may be different to that of other people transitioning. This aspect of the findings could inform the implementation of recommendations that people with KS/XXY and gender dysphoria “should then attend a respective specialist within a multidisciplinary setting” (Zitzmann et al., 2021, p. 157) and for “the set‐up of multidisciplinary centres or structures to care for patients with KS” (Zitzmann et al., 2021, p. 158). If gender identity services were available within endocrinology departments and KS/XXY clinics, people would not need to be referred out and care could be tailored to meet the specific needs of people with KS/XXY. This is presently the case at the KS multidisciplinary clinic at Guys and St Thomas's Hospital, London, UK, which could provide a model for other KS/XXY centers.

#### 5.1.5 Genetic counseling

The findings also have implications for genetic counseling, which is an important part of multidisciplinary care for KS/XXY (Espehana & Tomlinson, 2021). The potential to preserve fertility through intracytoplasmic sperm injection (ICSI) increased the relevance of genetic counseling for people with KS/XXY (Kruse et al., 1998) and one study concluded that genetic counseling should focus on early diagnosis and timely treatment for infertility (Zhang et al., 2015). However, genetic counseling can also focus on optimizing the maturation and well‐being of children with KS/XXY and helping adults with KS/XXY understand the syndrome and manage their concerns (FDNA Health, 2024; Espehana & Tomlinson, 2021; Howell & Grand, 2020). A previous study concluded that “genetic counseling can be enhanced for individuals with XXY and their families by providing accurate, up‐to‐date information, acknowledging the psychosocial impact of living with XXY, preparing individuals and families for potential challenges, and facilitating effective coping,” and that “genetic counselors may play an integral role as advocates to raise awareness about XXY among other health care providers” (Turriff et al., 2017, p. 736). Our findings provide insights that can extend the range of issues that genetic counselors discuss with people with KS/XXY and advocate on their behalf with other healthcare professionals.

### 5.2 Methodological strengths and limitations

The sample was homogenous in that all the participants had been diagnosed as KS/XXY, although participants varied in gender identity and country of residence. Because the study was advertised to potential participants as being an exploration of gender identity, gender diverse people with KS/XXY were probably over‐represented by comparison with the wider population of KS/XXY people. The sample was also predominantly White and middle class, so more research is needed on experiences of gender identity among people with KS/XXY among minority ethnic and disadvantaged socio‐economic groups.

As noted in the analytic strategy, the themes were developed to tell a story about participants' gender identity journeys. We stayed as close as possible to participants' experiences as they described them, and we interpreted them with the aim of giving participants a voice, shaping their described experiences into a narrative that could contribute to the development of more patient‐centered, evidence‐based care, which must have influenced the process of interpretation and meaning‐making. However, the data analysis followed analytic practices associated with the ontological (critical realist) and epistemological (phenomenological) approaches we adopted, which were consistent with experiential reflexive thematic analysis: “… experiential TA (including reflexive TA when used in experiential orientations) is concerned with exploring the truth or truths of participants' contextually situated experiences, perspectives, and behaviors. It is typically underpinned by some form of realist (naïve and critical) ontology […] and […] epistemologies including […] phenomenology” (Braun & Clarke, 2022a, p. 8).

### 5.3 Research implications

This was the first systematic qualitative exploration to our knowledge of how people with KS/XXY experienced gender identity and gender questioning and it was also the first time some participants had talked openly about the challenges they encountered regarding gender identity. Further research might include more in‐depth exploration of specific sub‐groups within the KS/XXY community, such as people during and after transitioning from male to female, to understand this process better and be able to offer better support. Also, longitudinal studies would be informative about the change process and critical influences as people negotiate their gender identities. Applying the concept of gender dysphoria for people with intersex conditions has been challenged (Davy & Toze, 2018), but future research could examine how people with KS/XXY experience gender diversity by comparison with other specific groups, such as people with autism (Cooper et al., 2022). Studies of doctors' and other healthcare professionals' attitudes to and perceptions of KS/XXY people and the factors that affect treatment decisions would help to understand and improve the treatment process. Research is also needed on how testosterone therapy impacts on a KS/XXY individual's gender identity, especially if they experience gender questioning or gender dysphoria prior to starting TRT. Trials of new procedures at endocrinology/andrology and Klinefelter syndrome clinics will also be needed to assess any benefits and provide an evidence base for new models of treatment. The present study focused on adult participants, but future research might also explore the ways that conversations about gender identity might be managed with children and young people and their families.

### 5.4 Conclusions

The study provided novel insights into the gender identity journeys of people with KS/XXY, from early attempts to understand and make sense of gender, through dealing with social pressures, questioning gender identities, developing gender identities more congruent with feelings, and experiences of hormone replacement therapy. These new insights can inform improved treatment and care for KS/XXY people.

## AUTHOR CONTRIBUTIONS

Claire Harkin conceived and designed the study, collected the data, led the data analysis, and wrote the first draft of the manuscript. James Elander contributed to the conception, design, methods, data analysis, and drafting of the manuscript. Claire Harkin and James Elander both made substantial contributions to the conception and design of the work, the interpretation of the data, and drafting the work and revising it critically for important intellectual content. James Elander and Claire Harkin confirm that they had full access to all the data in the study and take responsibility for the integrity of the data and the accuracy of the data analysis. Both authors gave final approval of the version to be published and agreed to be accountable for all aspects of the work in ensuring that questions related to the accuracy or integrity of any part of the work are appropriately investigated and resolved.

## CONFLICT OF INTEREST STATEMENT

Claire Harkin and James Elander declare that they have no conflicts of interest.

## ETHICS STATEMENT

Human studies and informed consent: Approval to conduct this human subjects research was obtained by the University of Derby Research Ethics Committee (Reference ETH2122‐1547). All procedures followed were in accordance with the ethical standards of the British Psychological Society and with the Helsinki Declaration of 1975, as revised in 2000. Informed consent was obtained from all participants included in the study. No non‐human animal studies were carried out by the authors for this article.

## Supporting information


Appendix S1

## Data Availability

The data that support the findings of this study are available from the corresponding author upon reasonable request.
