# Supplementary material for: A qualitative exploration of experiences of gender identity and gender questioning among adults with Klinefelter syndrome/XXY
Source: J Genet Couns. 2024 Jul 22;34(2):e1952. doi: 10.1002/jgc4.1952 (PMC11907177; doi:10.1002/jgc4.1952)
Supplement: Supplementary file 1 — Appendix S1 [file JGC4-34-0-s001.docx]

**Appendix S1. Interview Schedule**

**Welcome:**

- Welcome and thank participant for agreeing to take part in the study
- Ask how the participant would like to be addressed and what pronouns they would like me to use
- Inform the participant of my pronouns
- Talk through the Participant Information Sheet
- Review Consent Form
- Explain the debrief process and where they can go for additional support if required
- Ask if they have any questions before the interview begins

**Interview questions:**

1. Can you tell me when you were first diagnosed with 47 XXY/Klinefelter’s Syndrome (XXY/KS)?
2. Can you tell me how you were diagnosed?
3. What gender do you identify with, if any?
4. Have you ever felt confusion or dissatisfaction with your gender identity?
5. If so, how did it feel?
6. What resources or support have you been able to access in relation to any confusion or dissatisfaction you may have had about your gender identity?
7. Have these resources/supports been helpful or unhelpful?
8. How have you felt about your gender identity since your diagnosis?
9. Do you receive Hormone Replacement Therapy (HRT)?
10. If so, what kind?
11. How long have you been receiving HRT for? (If applicable)
12. How have you felt about your gender identity since you started HRT? (If applicable)
13. Have you received/been offered any psychological support since your diagnosis?
14. Have you received/been offered any psychological support since you started HRT? (If applicable)
15. What services or support would you like to see made available to help meet your needs or anyone else’s living with XXY/KS who may be experiencing gender identity confusion or dissatisfaction?
16. Is there anything else you would like to add?

**Debrief:**

· Talk through the Debrief Form

· Highlight supportive resources available should the participant require any
